# Youth cardiorespiratory fitness: evidence, myths and misconceptions

**DOI:** 10.2471/BLT.18.227546

**Published:** 2019-09-03

**Authors:** Neil Armstrong, Jo Welsman

**Affiliations:** a Children’s Health and Exercise Research Centre, St Lukes Campus, University of Exeter, Heavitree Road, Exeter, EX1 2LU, England.

## Abstract

Rigorously determined peak oxygen uptake is internationally recognized as the criterion measure of youth cardiorespiratory fitness. The assessment and interpretation of children’s and adolescents’ peak oxygen uptake and the relationship of the measure with other health-related variables are well documented. There has been a recent resurgence of interest in the prediction of peak oxygen uptake from field performance tests in young people. However, coupled with ratio-scaling of data and the raising of clinical red flags, these practices risk clouding our understanding of youth cardiorespiratory fitness and its relationship with current and future health. We believe these methods have the potential to mislead clinical practice and misguide recommendations for the promotion of youth cardiovascular health. We discuss relevant scientific evidence and interpretations that have emerged from predicting youth cardiorespiratory fitness from performance test scores. We argue that children deserve to have health care founded on evidence-based science and not on myths and misconceptions.

## Résumé

La consommation maximale d'oxygène rigoureusement déterminée est reconnue à l'échelle internationale comme la mesure du critère de la capacité cardiorespiratoire des jeunes. On dispose de nombreuses informations sur l'évaluation et l'interprétation de la consommation maximale d'oxygène des enfants et des adolescents, ainsi que sur le lien de la mesure avec d'autres variables relatives à la santé. On a récemment constaté un regain d'intérêt à l'égard de la prédiction de la consommation maximale d'oxygène à partir de tests de performance sur le terrain chez les jeunes. Néanmoins, associées à une mise à l'échelle proportionnelle des données et à la diffusion d'alertes cliniques, ces pratiques risquent de brouiller notre compréhension de la capacité cardiorespiratoire des jeunes et de son lien avec leur santé actuelle et future. Nous pensons que ces méthodes sont susceptibles d'altérer les pratiques cliniques et les recommandations liées à la promotion de la santé cardiovasculaire des jeunes. Nous examinons les données et les interprétations scientifiques pertinentes qui ont émergé de la prédiction de la capacité cardiorespiratoire des jeunes à partir des résultats des tests de performance. Nous soutenons que les enfants méritent de bénéficier de soins de santé fondés sur des données scientifiques, et non sur des mythes et des idées fausses.

## Resumen

La rigurosa determinación del consumo máximo de oxígeno es reconocida internacionalmente como la medida de criterio de la condición cardiorrespiratoria de los jóvenes. La evaluación e interpretación del consumo máximo de oxígeno de niños y adolescentes y la relación de la medida con otras variables relacionadas con la salud están bien documentadas. Recientemente ha resurgido el interés por la predicción del consumo máximo de oxígeno a partir de pruebas de rendimiento en campo en personas jóvenes. Sin embargo, junto con los datos de la escala de razón y el incremento de las señales de alerta clínicas, estas prácticas corren el riesgo de nublar nuestra comprensión de la condición cardiorrespiratoria en los jóvenes y su relación con la salud actual y futura. Se considera que estos métodos pueden inducir a error a la práctica clínica y a las recomendaciones mal orientadas para la promoción de la salud cardiovascular en los jóvenes. Se analizan las evidencias científicas relevantes y las interpretaciones que han surgido de la predicción de la condición cardiorrespiratoria en los jóvenes a partir de los puntajes de las pruebas de rendimiento. Se argumenta que los niños merecen que el cuidado de la salud se base en la ciencia basada en la evidencia y no en mitos y conceptos erróneos.

## ملخص

يُعرف معدل الامتصاص الأقصى للأوكسجين المحدد بشكل صارم بأنه مقياس معياري للياقة القلبية التنفسية لدى الشباب. تم توثيق كل من تقييم وتفسير معدل الامتصاص الأقصى للأوكسجين لدى الأطفال والمراهقين، وكذلك علاقة المقياس بالمتغيرات الأخرى ذات الصلة بالصحة. عاود الظهور مؤخراً الاهتمام بالتنبؤ بمعدل الامتصاص الأقصى للأكسجين من اختبارات الأداء الميداني لدى الشباب. ومع ذلك، وبالإضافة إلى زيادة نسبة البيانات وظهور أجراس إنذار سريرية، فإن هذه الممارسات تخاطر بالتأثير على فهمنا للياقة القلبية التنفسية لدى الشباب، وعلاقتها بالمؤشرات الصحية الحالية والمستقبلية. نحن نؤمن بأن هذه الطرق لديها القدرة على إفساد الممارسة السريرية وتشتيت التوصيات الخاصة بتعزيز صحة القلب والأوعية الدموية لدى الشباب. نحن نناقش الأدلة العلمية والتفسيرات ذات الصلة والتي ظهرت نتيجة توقع اللياقة القلبية التنفسية لدى الشباب بناء على نتائج اختبار الأداء. نحن نؤكد أن الأطفال يستحقون الحصول على رعاية صحية ترتكز على العلوم المبنية على الأدلة وليس على الأساطير والمفاهيم الخاطئة.

## 摘要

严格确定的峰值摄氧量是国际公认的青少年心肺健康标准。儿童与青少年的峰值摄氧量的评估和解释以及该量值与其他健康相关变量的关系已有详细记录。最近年轻人对于通过现场表现测试来预测峰值摄氧量的兴趣再次高涨。然而，伴随着数据比值缩放和临床危险信号的增加，这些做法使我们对青少年心肺健康及其与当前和未来健康的关系认识不清。我们认为这些方法有可能会把临床实践引入误区并且误导有关青少年心血管健康的建议。我们讨论了通过表现测试评分预测青少年心肺健康中出现的相关科学证据和解释。我们主张儿童应该享有以循证科学为基础，而不是基于误区和误解的医疗保健。

## Резюме

Тщательно определяемое пиковое потребление кислорода считается международным критерием определения кардиореспираторной выносливости у молодежи. Оценка и интерпретация пикового потребления кислорода у детей и подростков и связь этого параметра с другими переменными, относящимися к здоровью, хорошо зафиксированы документально. Наблюдается возрождение интереса к прогнозированию пикового потребления кислорода при проведении функциональных проб среди молодежи. Однако в сочетании с масштабированием данных и появлением сомнений клинического характера эти практики могут осложнить понимание кардиореспираторной выносливости молодежи и ее связи с текущим и будущим здоровьем. Авторы полагают, что эти методы могут быть обманчивыми для клинической практики и послужить основой для неправильных рекомендаций по профилактике сердечно-сосудистых заболеваний среди молодежи. Авторы обсудили соответствующие научные обоснования и интерпретации, которые появились в связи с прогнозированием кардиореспираторной выносливости молодежи по результатам функциональных проб. Авторы утверждают, что развитие системы детского здравоохранения должно опираться на научно обоснованные факты, а не на мифы и искаженные представления.

## Introduction

Cardiorespiratory fitness defines the ability of the body to deliver oxygen from the atmosphere to the skeletal muscles and to use it to generate energy to support muscle activity during exercise. Peak oxygen uptake is internationally recognized as the gold standard measure of youth cardiorespiratory fitness. The assessment and interpretation of peak oxygen uptake and its evidence-based relationship with health-related variables are extensively documented.^1^ However, data from field tests of performance, inappropriate scaling of peak oxygen uptake and the current trend for identifying individuals alleged to be in need of intervention have clouded our understanding of youth cardiorespiratory fitness, and its relationship with children’s current and future health.^2^^–^^4^ We believe that flawed assessments, and unsound interpretations of cardiorespiratory fitness have led to the development of myths and misconceptions which may impact adversely on children’s health care.

## Evidence base

The first laboratory investigation of youth physical fitness was reported in 1938. Cardiorespiratory fitness, as represented by peak oxygen uptake, has subsequently become one of the most studied physiological variables in the history of paediatric exercise science.^5^

### Assessment of cardiorespiratory fitness

In over 80 years of intensive investigation, the assessment of youth peak oxygen uptake has been progressively developed and refined as new technology has been introduced into paediatric exercise science laboratories. The measurement of youth peak oxygen uptake has been comprehensively reviewed elsewhere.^6^^–^^8^ Topics covered include a critical examination of exercise test protocols; techniques of measuring exercise intensity; apparatus used to collect respiratory gases; size of components of respiratory gas collection systems; respiratory gas sampling intervals; and the criteria for maximal effort during exercise. Reviewers have emphasized that the methods and apparatus used should be carefully reported for comparative purposes. In our laboratory we have calculated that the typical error of measurement of youth peak oxygen uptake is about 4% across three tests each a week apart.^9^

While rigorous determinations of peak oxygen uptake have high reliability, caution is needed when data need to be compared across laboratories. Peak oxygen uptake is routinely determined with the study subject running on a treadmill or pedalling on a cycle ergometer. Due to the greater exercising muscle mass, enhanced venous return, higher stroke volume and reduced peripheral resistance during running, treadmill-determined values are around 11–14% higher than those determined on a cycle ergometer.^10^ Yet some laboratories pool treadmill and cycle ergometer values^11^ or apply fixed correction factors to accommodate lower cycle ergometer values of peak oxygen uptake.^12^ These values are then used to establish age-related cut-off points for cardiometabolic health and future risk of cardiovascular disease in individuals. However, pooling data in this way is a confounding factor in interpretation of the data, as differences between peak oxygen uptake values determined by treadmill and cycle ergometer vary widely with age and maturity status. We argue that this practice of pooling data from different exercise modes should cease.^10^

### Development of cardiorespiratory fitness

Peak oxygen uptake is often expressed in relation to age or body mass,^13^ but it is simplistic to describe it in this manner. Peak oxygen uptake increases in accordance with morphological and physiological changes related to growth and maturation. The timing and tempo of these changes are specific to individuals.^1^^,^^13^ Defining credible norms for age- or body mass-related cardiorespiratory fitness is therefore not feasible, regardless of whether peak oxygen uptake is expressed in absolute terms (as L per min) or, as is often the case, in ratio with body mass (as mL per kg body mass per min).^8^ The most powerful morphological influence on peak oxygen uptake is not body mass but fat-free mass.^13^ Increases in fat mass do not influence the development of peak oxygen uptake.^14^

Boys’ peak oxygen uptake values are higher than those of girls, at least from late childhood, and this difference increases as children progress through adolescence, reaching about 40% higher in post-pubertal 18-year-old boys.^15^ The introduction of non-invasive technologies to the study of developmental exercise physiology has stimulated research into the mechanisms underlying peak oxygen uptake. Studies using Doppler echocardiography have indicated that the small pre-pubertal sex difference in peak oxygen uptake, around 10%, can be largely attributed to greater stroke volume in boys. Whether this difference is due to differences in cardiac size^16^ or cardiac function^17^ is contentious. In contrast, a study using thoracic bioelectrical impedance and magnetic resonance imaging reported that the sex difference observed in peak oxygen uptake was due to maximal arteriovenous oxygen differences, with no significant sex difference in maximal stroke volume or resting heart size.^18^ A study using near-infrared spectroscopy reported a poorer matching of muscle oxygen delivery to oxygen utilization in girls compared with boys and suggested that this difference may contribute to sex differences in peak oxygen uptake.^19^ Further research is required to fully understand the underlying mechanisms.

Boys’ marked increase in fat-free mass (reflecting increases in muscle mass) accounts for most of the progressive sexual divergence in peak oxygen uptake after puberty.^13^ Driven by maturation, fat-free mass increases by about 40% and 90% in girls and boys, respectively, from 11–16 years of age.^20^ The great majority (about 83%) of the increase in fat-free mass in boys takes place over a 4-year period, centred on the time of peak height velocity. The greatest increase in girls’ fat-free mass (about 31%) occurs over a shorter 2-year period, centred on peak height velocity, and then levels off in accordance with the development of peak oxygen uptake.^20^ Boys’ peak oxygen uptake may be increased further by a sex-specific increase in haemoglobin concentration in the late teenage years, which enhances oxygen-carrying capacity of the blood in boys. This theory has yet to be empirically demonstrated in longitudinal studies.^21^ We have published a detailed analysis of the development and assessment of peak oxygen uptake elsewhere.^6^

### Physical activity and cardiorespiratory fitness

To explain relationships between physical activity and cardiorespiratory fitness we first need to differentiate between habitual physical activity and exercise training. Habitual physical activity has been defined as “the usual physical activity carried out in normal daily life in every domain and any dimension.”^22^ Exercise training consists of a planned, structured exercise programme that is sustained for an adequate length of time, with sufficient intensity and frequency to induce changes in components of physical fitness. Cardiorespiratory fitness, physical activity behaviour and exercise trainability are all heritable traits. However, discussion of genetics and molecular paediatric exercise physiology are outside the remit of the present paper and interested readers are referred to a review article published elsewhere.^23^

Different methods of assessing habitual physical activity are not always comparable,^22^ but studies consistently show that boys are more active than girls and that physical activity decreases with age in both sexes. The number of young people reported to satisfy current physical activity guidelines varies across studies. The International Olympic Committee Consensus Statement on the health and fitness of young people through physical activity and sport suggests that when objective methods of measurement (such as accelerometry) are used less than 25% of young people meet current physical activity guidelines.^24^

A systematic review of the literature^25^ found and analysed 69 training studies of youths 8–18-years of age. The review noted that rigorously designed training studies are consistent in demonstrating that appropriate training increases youth peak oxygen uptake, irrespective of sex, age or maturity status. Collectively, the data show that three 20 minute sessions per week of continuous intensity training at approximately 85–90% of maximum heart rate, or high-intensity interval training at around 95% of maximum heart rate interspersed with short recovery periods, will induce on average an 8–9% increase in youth peak oxygen uptake in 10 to 12 weeks. Investigations based on lower exercise intensities (but still higher than those recommended in current health-related physical activity guidelines) have been showed to be ineffective in improving cardiorespiratory fitness.^25^

Studies stretching back over 45 years have consistently demonstrated that there is no meaningful relationship between peak oxygen uptake, as determined by rigorous methods, and objectively monitored habitual physical activity in youth.^26^ For more information, readers can consult our review of the published studies to date.^26^ These data have been confirmed by longitudinal investigations. One study monitored 202 children (98 girls) and used multilevel modelling to examine age, maturity status and morphological influences on habitual moderate and vigorous physical activity from 11 to 13 years of age.^27^ Having controlled for the primary variables, the researchers introduced peak oxygen uptakeand found that the models revealed no significant relationship with habitual physical activity. The investigators then analysed peak oxygen uptake in relation to accumulated time spent in at least moderate intensity physical activity. This analysis showed that even when controlling appropriately for body mass, peak oxygen uptake increased with age, whereas habitual physical activity decreased with age in both sexes. This finding is consistent with the existing literature on both physical activity^26^ and cardiorespiratory fitness.^6^ Similarly, after analysing 23 years of data, researchers at the Amsterdam Growth and Health Study concluded that there was no association between habitual physical activity and peak oxygen uptake in either males or females.^28^

The lack of a meaningful relationship between habitual physical activity and peak oxygen uptake is not surprising as young people rarely if ever experience the intensity and duration of physical activity necessary to increase their cardiorespiratory fitness. These findings do, however, seriously challenge recent proposals that physical activity interventions can be evaluated by changes in peak oxygen uptake estimated from performance tests.^2^

## Myths and misconceptions

Scientists have been aware of the limitations of performance tests in predicting cardiorespiratory fitness for over 50 years. Typical comments include: “in the average child scoring in the performance tests is largely dependent on body size, and this series of tests is of no help in predicting working capacity or aerobic capacity”^29^ and “the performance test may merely be a complicated method of identifying tall or fat pupils.”^30^ We have shared these concerns with the academic community for over 30 years. In 1988, we published an evaluation of the 20m shuttle-run test in 11–14-year-old boys and reported a common variance of 29% between performance in the test and rigorously determined peak oxygen uptake. We concluded that use of the test could not be supported as a valid substitute for a direct determination of peak oxygen uptake.^31^

At the time, we assumed that performance tests would cease to be used in scientific research, owing to the development of online breath-by-breath analysis systems, new technologies (such as mass spectrometry and telemetry) and sophisticated statistical modelling techniques. On the contrary, interest in performance tests has been revived, particularly in estimating peak oxygen uptake from 20m shuttle-run test scores. Scores collated from over a million children with data collected from different countries with varying cultures have been used to estimate peak oxygen uptake and produce international cardiorespiratory fitness norms^32^ and cross-country comparisons of who are the fittest children.^33^ Scores from children as young as 2 years have been converted into so-called reference standards for preschool children.^34^ Moreover, and of serious concern to us, is that performance in 20m shuttle-run tests has been recommended to evaluate physical activity interventions;^2^ to establish European normative values for fitness and health profiling;^35^ to survey and monitor international health and fitness;^36^ to determine metabolic and cardiovascular risk;^37^ and to identify individual children who warrant intervention to improve their current and future health.^4^

### Shuttle-run test

The 20m shuttle-run test is not a measure of cardiorespiratory fitness, but a function of the willingness and capability of individuals to run between two lines 20m apart while keeping pace with audio signals, which require the running speed to increase each minute. Participants run in groups until they are unwilling or unable to continue and the number of shuttles completed is converted into an estimate of peak oxygen uptake through a prediction equation. There are at least 17 different prediction equations currently in use to estimate peak oxygen uptake from 20m shuttle-run test scores, resulting in substantially different estimates of peak oxygen uptake.^32^ A recent meta-analysis of published studies revealed that 51% (18/35) of the correlation coefficients between test scores and youth peak oxygen uptake explained less than 50% of the total variance in peak oxygen uptake. The authors concluded that criterion validity was only moderate and “testers must be aware that the performance score of the 20 metre shuttle-run test is simply estimation and not a direct measure of cardiorespiratory fitness.”^38^

A recent review^39^ reported that peak oxygen uptake can only be estimated to within ± 10 mL per kg per min from the 20m shuttle-run but, as this represents around 20–25% of typical values, the limitations of the test are clear. Similarly, the poor test–retest reliability of the test is reflected by 95% confidence intervals of ± 2.5 stages on tests lasting four to six stages.^40^ Large sex differences in performance on the test are common, but in some countries the reported unexplained sex differences in the performance of teenagers are as high as 95–100%,^41^ which is more than double the true sex difference in cardiorespiratory fitness. If, in some cultures teenage girls are less willing than boys to publicly run 20m shuttles until they are truly exhausted, published international norms based on test performance are compromised.

Flawed methods lead to misleading interpretations. A prime example is the assertion that there has been a “substantial decline in cardiorespiratory fitness since 1981, which is suggestive of a meaningful decline in population health.”^42^ This statement was based on collations of cross-sectional 20m shuttle-run test estimations of peak oxygen uptake. In direct contrast, compilations of international peak oxygen uptake data over a similar timeframe present no compelling evidence that youth cardiorespiratory fitness has declined.^24^^,^^43^^–^^45^ We hold a substantial published database of cardiorespiratory fitness measures for youths 9–18 years of age in the United Kingdom of Great Britain and Northern Ireland, covering a period of over 30 years, with over 3000 rigorous laboratory determinations of peak oxygen uptake.^3^^,^^13^ We can confirm that, at least since 1985, there has been no discernible change in the cardiorespiratory fitnessof boys and girls from the same catchment area and schools.

According to advocates of the 20m shuttle-run test, the explanation for this alleged decline in cardiorespiratory fitness is that there has been a large temporal increase in youth fatness. Researchers have claimed that “direct analysis of the causal fitness–fatness connection indicates that increases in fatness explain 35–70% of the declines in cardiorespiratory fitness*.”*^32^ As fat is largely metabolically inert and does not influence cardiorespiratory fitnesss^14^ there is no causal fitness–fatness connection. Carrying extra fat mass over a series of 20m shuttle-runs does, however, increase the individual’s work in each shuttle and adversely affect their performance on the test. This flaw in data interpretation is compounded further by 20m shuttle-runs test estimates of peak oxygen uptake being expressed in ratio with body mass (in mL per kg per min) and therefore including fat mass in the denominator.

### Ratio-scaling

The fallacy of ratio-scaling peak oxygen uptake was demonstrated 70 years ago.^46^ Expressing cardiorespiratory fitness as ratio-scaled peak oxygen uptake favours lighter youth (for example, clinically underweight or delayed maturing) and penalizes heavier youth (for example, overweight or advanced maturing). Tutorial papers and recent cross-sectional and longitudinal analyses of over 2000 treadmill determinations of peak oxygen uptake have demonstrated theoretically and empirically that there is neither a sound scientific rationale nor a statistical justification for ratio-scaling of youth peak oxygen uptake.^3^^,^^10^^,^^13^^,^^47^

Widespread, erroneous use of ratio-scaling has clouded the understanding of youth cardiorespiratory fitness. Ratio-scaled peak oxygen uptake data indicate that boys’ cardiorespiratory fitness is stable from 10–18 years of age and girls’ values progressively decline with age. Whereas when researchers control appropriately for body mass there is a progressive increase in peak oxygen uptake with age in both sexes.^13^ Moreover, ratio-scaled data misinterpret true relationships between cardiorespiratory fitness and indicators of health.^3^^,^^46^^,^^48^^,^^49^ A topical example is reporting correlations between cardiovascular risk factors and ratio-scaled peak oxygen uptake in overweight and obese youth, when any association is more likely to reflect overweight or obese status than cardiorespiratory fitness.^48^ A recent systematic review highlighted how many articles relating youth cardiorespiratory fitness to health *“*did not account for important confounding factors such as adiposity.”^49^ For example, higher peak oxygen uptake in ratio with body mass was associated with lower body fatness but there was no relationship between the two variables when peak oxygen uptake was not expressed in ratio with body mass. Similarly, a higher peak oxygen uptake appeared to be associated with a lower ratio of total cholesterol to high-density lipoprotein cholesterol values but, again, the association was only present when peak oxygen uptake was expressed in ratio with body mass.^49^ A published comment on the review pointed out that, in addition, the impact of body mass on performance in field tests with low validity and poor reliability differs across tests and may also affect the size of the alleged associations with health outcomes.^50^

### Clinical red flags

The relationship between cardiorespiratory fitness and health is confounded further by the emergence and growing popularity of so-called clinical red flags that “identify children and adolescents who may benefit from primary and secondary cardiovascular prevention programming.”^4^ Estimated peak oxygen uptake values for children, adolescents and young adults (8–18 years of age) below 42 and 35 mL per kg per min for males and females, respectively, are identified as raising a clinical red flag.^4^ Cardiorespiratory fitness develops in accordance with sex, age and maturation and a range of morphological and physiological covariates whose timing and tempo of changes are specific to individuals.^1^^,^^13^ We believe that classifying pre-pubertal, pubertal and post-pubertal youth on the basis of a single value of peak oxygen uptake in ratio with body mass is therefore unjustifiable. Moreover, when peak oxygen uptake is predicted from a test with validity, reliability and cultural problems the measure becomes indefensible.

## Conclusions

Rigorous laboratory assessment of peak oxygen uptake is a well-established approach but there is currently no valid and feasible method of evaluating youth cardiorespiratory fitness at the population level. We argue that the estimation of youth cardiorespiratory fitness from performance tests such as the 20m shuttle-run test is untenable. Also, we challenge the use of per body mass ratio-scaling to investigate relationships of cardiorespiratory fitness with health-related variables; the use of age-related norms; the designation of clinical red flags; and the use of performance test estimates of cardiorespiratory fitness to evaluate physical activity interventions.

Scientists have an ethical responsibility to ensure that the methods underpinning their research are fit for purpose. They also have a moral responsibility to ensure that data interpretation is sound. Published papers continue to make interpretations of youth peak oxygen uptake not founded on rigorous scientific evidence and shown repeatedly and extensively to be flawed. The dissemination of such data is likely to misinform clinical practice, mislead policy statements and misguide recommendations designed to promote youth health.
